# VIGOR, an annotation program for small viral genomes

**DOI:** 10.1186/1471-2105-11-451

**Published:** 2010-09-07

**Authors:** Shiliang Wang, Jaideep P Sundaram, David Spiro

**Affiliations:** 1 J. Craig Venter Institute, 9704 Medical Center Drive, Rockville, MD 20850, USA; 2 Computational Genomics Laboratory, Department of Biology, Georgetown University Georgetown, Washington DC 20057, USA

## Abstract

**Background:**

The decrease in cost for sequencing and improvement in technologies has made it easier and more common for the re-sequencing of large genomes as well as parallel sequencing of small genomes. It is possible to completely sequence a small genome within days and this increases the number of publicly available genomes. Among the types of genomes being rapidly sequenced are those of microbial and viral genomes responsible for infectious diseases. However, accurate gene prediction is a challenge that persists for decoding a newly sequenced genome. Therefore, accurate and efficient gene prediction programs are highly desired for rapid and cost effective surveillance of RNA viruses through full genome sequencing.

**Results:**

We have developed VIGOR (Viral Genome ORF Reader), a web application tool for gene prediction in influenza virus, rotavirus, rhinovirus and coronavirus subtypes. VIGOR detects protein coding regions based on sequence similarity searches and can accurately detect genome specific features such as frame shifts, overlapping genes, embedded genes, and can predict mature peptides within the context of a single polypeptide open reading frame. Genotyping capability for influenza and rotavirus is built into the program. We compared VIGOR to previously described gene prediction programs, ZCURVE_V, GeneMarkS and FLAN. The specificity and sensitivity of VIGOR are greater than 99% for the RNA viral genomes tested.

**Conclusions:**

VIGOR is a user friendly web-based genome annotation program for five different viral agents, influenza, rotavirus, rhinovirus, coronavirus and SARS coronavirus. This is the first gene prediction program for rotavirus and rhinovirus for public access. VIGOR is able to accurately predict protein coding genes for the above five viral types and has the capability to assign function to the predicted open reading frames and genotype influenza virus. The prediction software was designed for performing high throughput annotation and closure validation in a post-sequencing production pipeline.

## Background

Rapid and cost effective genomic surveillance of RNA viruses is a critical component of vaccine and drug development pipelines for the control of emerging viral diseases. Improvements in sequencing technology and the concomitant decrease in costs have made it easier and more common for the re-sequencing of large genomes as well as parallel sequencing of small genomes. This has led to an exponential increase in the genomic data available in public databases. However, accurate gene prediction is a challenge that has created a bottleneck in the gene predication pipeline.

Two major approaches, *ab initio *gene finding and similarity-based prediction [1], have been commonly applied to gene prediction. The *ab initio *method, also known as the intrinsic statistical strategy, computes statistical data such as the nucleotide frequencies and their ordering in a set of genomic sequences that have been characterized. This is because the nucleotide frequencies and ordering for each genome usually differ between protein coding and non-coding regions. However, viral genomes, because of their small genome sizes, may not provide sufficient training data to derive the parameters necessary to attain the best performance possible for this approach. The heuristic method, which determines the parameters of the necessary models from short sequences, was adopted by several gene prediction programs, e.g., GeneMarkS [2]. Small amount of genomic sequence, but long enough to produce the efficient Markov models, usually a small fraction of large genome or small genomes like viral genomes, is needed for this method. The linear function reflecting the relationship between the nucleotide frequencies in the three codon positions and the global nucleotide frequencies is obtained by analyzing the small amount of DNA sequence. These derived data will be used to predict protein coding genes by the heuristic method [3]. The *ab initio *method uses these trained or self-trained modules to select the protein coding regions and predict coding sequences. The similarity-based method predicts protein coding sequences by a different strategy, identifying gene coding sequences by sequence similarity alignment to reference sequences which are closely related evolutionarily. Since these two approaches use different strategies to detect the protein coding sequences, the performances are different and depend on the training data set and reference sequence data. Usually, *ab initio *approach is more sensitive than similarity-based approach, while the performance of similarity-based method has greater specificity. This is because *ab initio *methods predict some false positive exons and genes in intergenic regions and introns, while similarity-based tools cannot detect genes if the homologous sequences are not included in the reference data.

Although most viral genomes are relatively small compared to eukaryotic and prokaryotic genomes, the gene structure of viral genomes can be complex. For example, introns, alternative splicing, overlapping genes, and ribosomal slippage exist in many viral genomes. Thus an all purpose gene finder cannot be easily adapted for gene prediction across all virus families. However, if the genome scaffold and the gene features of a viral genome are well understood, a similarity-based gene prediction approach based on the curated gene repertoire for a specific virus genus with attention to particular recognition features, such as, splice sites and mature peptide cleavage sites can be adapted, and perform better than an *ab initio *gene finder.

The National Institute of Allergy and Infectious Diseases (NIAID) funds a Genomic Sequencing Center for Infectious Diseases (GSCID) at the J. Craig Venter Institute (JCVI). One of the goals of the GSCID is high throughput sequencing of various viral pathogens. The viral genome sequencing projects at JCVI have resulted in publication of more than 4000 influenza virus genomes from clinical and animal reservoir specimens, and hundreds of coronavirus and rotavirus sequences. Prediction of protein coding genes encoded in these viral genomes is a critical step to understanding these pathogenic viruses. In order to have a flexible, accurate gene prediction tool for utilization in high throughput viral genome sequencing projects, we developed a viral annotation program, VIGOR (Viral Genome ORF Reader). VIGOR uses a similarity-based approach to detect open reading frames (ORF) in various viral genomes by similarity searches against custom reference protein sequence databases. VIGOR takes into account differences between the genomic structures of viral taxonomic groups. VIGOR is tailored for the designated viruses with complex gene features such as splicing and frame-shifting, and it is able to predict genes accurately in influenza (group A, B, and C), coronavirus (including SARS coronavirus), rhinovirus, and rotavirus genomes. It was also designed to assign function to the predicted ORFs and genotype influenza viruses. In addition to gene prediction, VIGOR can also be used as a tool to validate sequence accuracy and completeness during the genome finishing process.

## Implementation

### 1. Custom protein databases

Complete protein sequences of all ORFs for influenza virus, rotavirus, rhinovirus and coronavirus subtypes were downloaded from GenBank, and redundant sequences were removed by custom scripts. For coronavirus and SARS coronavirus, both the orf1a polypeptide sequences and orf1b polypeptide sequences were included in the coronavirus and SARS coronavirus reference database.

### 2. Detection of protein coding regions in viral genomes

Similarity searches between viral genomic sequences and a custom protein database are conducted by BLASTX [4]. The longest aligned region detected by the similarity search spanning one single protein sequence plus 100 bases upstream and downstream sequences is selected as the potential coding region for the particular open reading frame. This region is then further searched for genomic features indicative of the coding region.

### 3. Identification of start codon and stop codon

A similarity search is performed again between the potential coding region and the custom protein database. The protein sequence with highest identity in the similarity search is established as the reference sequence for the identification of the start and stop codons. If the first codon in the potential coding sequence is ATG and aligns with the first residue in the reference sequence, this ATG is selected as the start codon; otherwise, the nearest upstream in-frame ATG is selected as the start codon. If no in-frame ATG is present in the upstream region of the aligned sequences, the 60 nucleotides downstream of the first aligned residue are scanned for the start codon. Sequences downstream of the last aligned residue of the potential coding sequence are scanned for in-frame stop codons (TAA, TGA, and TAG) and the closest stop codon to the last aligned residue is selected.

### 4. Selection of splice sites in influenza genomes and detection of ribosomal slippage sites in the first open reading frame of coronavirus genomes and SARS coronavirus genomes

Mature mRNA for the influenza M2 and NS2 genes is produced by internal splicing. The conserved splice donor and acceptor sites (GT...AG) [5] are scanned around the alignment joint sites between the gap and aligned regions. The splice sites which result in the best alignment between the translated protein and the reference sequence are selected. The two main criteria for the selection of splice sites are identity to reference sequence and sequence length of the translated protein; however, if these two do not agree with each other, sequence length has priority in choosing the final splice sites.

The first transcript of coronavirus genomes and SARS coronavirus genomes encodes two polyproteins because of ribosomal slippage during translation [6]. The first polyprotein (orf1a) is translated from the sequence with start and stop codons, and normal translation, while the synthesis of the second polyprotein (orf1ab) is dependent on a -1 nucleotide ribosomal frameshift induced by a "slippery" sequence of the type "UUUAAAC" upstream of the orf1a stop codon [7]. VIGOR examines the region upstream of the orf1a stop codon to map out precisely the "UUUAAAC" string. It then shifts back the reading frame by -1 nucleotide (from AAC to AAA) within the slippery sequence, and the -1 frame is extended to generate the coding sequence for the translation of orf1ab.

### 5. Genotyping of influenza virus

There are 16 subtypes for hemagglutinin protein (HA) and 9 subtypes for neuraminidase (NA) in group A influenza viruses, but only one subtype for HA and one subtype for NA for influenza B viruses [8,9]. The genotypes of influenza viruses can be categorized by the hemagglutinin protein (HA) and neuraminidase (NA). In the custom VIGOR database, HA and NA subtype sequences are stored and used to categorize the genotypes of these two influenza proteins based on the best similarity.

### 6. Identification of the mature peptide cleavage sites for the rhinovirus polyprotein and SARS coronavirus orf1a and orf1ab

The rhinovirus polyprotein is cleaved into 11 mature, functional peptides by proteases. There are conserved cleavage signature sequences for 9 cleavage sites [10]. In order to predict mature peptides, the polyprotein sequence is aligned with the sequences in VIGOR's custom rhinovirus mature peptide database to identify the mature peptide cleavage sites. In the absence of a conserved signature sequence, the putative cleavage site whose products result in best alignments for both sequence length and similarity is selected.

The conserved mature peptide cleavage signature sequences for the orf1a and orf1ab of SARS coronavirus derived from sequence comparative alignment and literature [11-13]. At the position P1 (the position just before the cleavage site) is the conserved Glutamine (Q), the signature residue recognized by papain-like proteinase. Mature peptide cleavage sites are determined by mature peptide length and conserved cleavage signature sequences.

### 7. Further criteria for gene prediction

(i) Coding sequences must have both start and stop codons, or span the 5' or 3'-end of the input sequences for partial genomic sequences.

(ii) There should not be an internal stop codon or frameshift in the coding sequences except for the orf1ab in coronavirus genomes and SARS coronavirus genomes.

(iii) The exon number must be the same as that of the homologous gene in the custom database.

(iv) The translated product of the coding region must span 95% of the length of the homologous protein unless the homolog in the reference database is shorter than 50 amino acids except influenza genomes. For influenza genes, if the encoded protein is 500 amino acids or longer (PB2, PB1, PA and HA), the translated product must span more than 98% of the length of the reference protein; The length difference between the translated product and the reference protein should be within 5 amino acids for NS2 and M2 genes (shorter than 150 aa); the encoded NS, MP, NP and NA proteins should not be 15 amino acid shorter than reference proteins.

(v) If any of these criteria is not observed, the sequence region homologous to reference sequences will be identified as a non-functional gene region, and will be marked as a possible sequence mutation.

### 8. Implementation

VIGOR is available at http://www.jcvi.org/vigor. Viral genomic sequences in either FASTA or multi-FASTA format can be pasted or uploaded from a local file into the input sequence area through a GUI interface which was implemented in Perl CGI, PHP and Javascript. VIGOR is supported by a multi-tiered backend service called Arcturus. This service includes the capability to receive and process all types of web form submissions. The submission component transforms the submitted data into a job and the queuing component schedules the job for processing. A post-processing component formats the results and notifies the submitter that the gene predictions can be viewed following the completion of the job.

Arcturus is responsible for invoking the appropriate gene prediction program in the VIGOR package for the specified virus type. Currently, all jobs are executed on a single, dedicated server. The backend service is implemented to support scalability. The entire backend service was implemented in Perl.

A user needs to select the virus type through a pull-down virus name menu prior to submitting the sequence data. The user will be informed of the link to download the prediction result by email following the VIGOR run. The output includes three files. The main output file is the gene prediction file which includes the predicted peptide sequence length, coordinates of the coding regions, splice sites if applicable, protein function and genotype if available, and the predicted amino acid sequences. The other two output files are the cDNA sequence file and a file of the alignment between the predicted protein and the best match in the custom database so that the user can evaluate the prediction. If mutations which generate internal stop codons or frameshifts are detected, the mutated sequences plus the flanking sequences will be presented in the output. The alignment data from the BLASTX search is also included in the gene prediction file.

## Results and Discussion

To assess the performance of VIGOR, five sets of annotated sample sequences were downloaded from GenBank. These included influenza virus, rotavirus, coronavirus, SARS coronavirus and rhinovirus. VIGOR was compared to three separate gene finding programs: GeneMarkS, ZCURVE_V and FLAN. GeneMarkS [2] and ZCURVE_V [14] both are *ab initio *universal gene finding programs. FLAN is a web-based gene prediction tool specific for influenza viruses that was developed at NCBI for the Influenza Genome Sequencing Project and has been widely used to annotate influenza sequences [15]. FLAN uses the similarity based approach, comparing the influenza genomic sequences with annotated influenza peptide sequences to identify open reading frames. GeneMarkS and ZCURVE_V were run for all sample genomes, while FLAN was run only on influenza genomes.

The results were evaluated by comparison between the gene finder predictions and annotations in GenBank. Sensitivity (Sn) and Specificity (Sp) were employed to evaluate the performance of the programs tested. Sn is defined as the percent correct predictions out of all annotated genes in the dataset. Sp is defined as the percent correct predictions out of all predictions. Correct predictions are those which are the same as the GenBank annotations. Any prediction that was not identical to the GenBank annotation was inspected manually by similarity searching against the NCBI non-redundant (NR) protein database. If the new prediction was highly similar (E value <1e-10) to a viral protein spanning 95% of the homologous protein length, it was categorized as a correct prediction. A partially correct prediction was assigned when the prediction overlapped with the GenBank annotation in the same reading frame but with a different start codon. An incorrect prediction was assigned when the prediction overlapped with the GenBank annotation but in a different reading frame, or cannot be validated by sequence similarity.

### Influenza

The influenza virus genome consists of eight RNA segments that encode one or two proteins each. Splicing is involved in the expression of the MP and M2 proteins from segment 7 (MP segment) in group A influenza viruses and NS1 and NS2 proteins from segment 8 (NS segment) in both group A and group B viruses [16]. Segment 2 (PB1 segment) encodes two proteins, PB1 and PB1-F2, in some influenza genomes. The coding sequence of PB1-F2 is completely embedded in the PB1 coding region with a different reading frame [17]. In order to test its accuracy in gene finding, 2376 full and partial influenza segment sequences including group A, group B, and group C viruses, encoding 3177 annotated proteins, were run through VIGOR. VIGOR predicted 3178 ORFs encoded by these segments. Among these predicted protein sequences, 99% (3169/3178) ORFs completely agreed with the annotations in GenBank. Three predicted ORFs were partially correct and one ORF was incorrectly predicted (Table 1).

**Table 1:** The annotations in GenBank and predictions by VIGOR, FLAN, GeneMarkS and ZCURVE_V of segmented RNA viruses, influenza viruses (Flu) and rotaviruses (Rtv)

		No. of seq.	No. of genes	No. of correct pred.	Sp^+^(%)/Sn^++^(%)	No. of partial correct gene	Discrepancy*	No. of missing genes	No. of mis-predicted genes	No. of new genes	Geno-typing
	Ref. Seq	2376	3177								
	
	VIGOR		3178	3169	99.40/99.9	3	5	0	1	0	Yes
	
Flu	FLAN		3149	3124	99.2/98.33	6	57	N/A	9	0	Yes
	
	GeneMarkS		2754	1119	40.63/35.22	1288		770	347	0	No
	
	ZCURVE_V		2809	2296	81.74/72.27	40		841	473	0	No

	Ref. Seq.	1166	1158								
	
	VIGOR		1202	1199	99.75/99.75	3		0	0	44	Yes
Rtv											
	GeneMarkS		1208	378	31.29/32.64	776		5	54	1	No
	
	ZCURVE_V		1171	1113	95.05/96.11	45		1	13	1	No

^+^. Specificity;^++ ^Sensitivity. *. Discrepancy cases between this prediction and GenBank annotation.

The influenza sequence set was also run through FLAN (influenza specific gene prediction program). The specificity and sensitivity of FLAN are quite comparable to VIGOR (Table 1). However, 47 genes annotated in GenBank were predicted as non-functional genes by FLAN because of different problems detected in these sequences including internal stop codons, frame shifts or incorrect splicing. These predictions were marked as discrepancies in Table 1. Nine small ORFs detected by FLAN were missing from the VIGOR prediction list; all of these were homologous to PB1-F2 protein. These PB1-F2 proteins predicted by FLAN were manually compared to the homologous PB1-F2 sequences. It was found that multiple stop codons are present in the region homologous to the N-terminal domain of PB1-F2. Although the annotations in GenBank for 6 of these nine sequences are same as FLAN predictions, most likely all these nine sequences encode non-functional PB1-F2 genes.

The same set of influenza genomic sequences was also run using GeneMarkS and ZCURVE_V, two *ab initio *approach gene prediction tools for viral genomes (Table 1). The specificity and sensitivity for GeneMarkS was 40.63% and 35.22% respectively; while the specificity and sensitivity for ZCURVE_V was 81.74% and 72.27%. Similar numbers of GenBank annotated genes were missed by both GeneMarkS (770 genes) and ZCURVE_V (841 genes). Manual inspection showed that the majority of the overlooked genes were PB1-F2, NS2 and M2 genes. Several studies have shown that embedded genes and splicing often pose problems for viral gene prediction algorithms [2,14]. For example, ZCURVE_V could not identify the *Tat *gene correctly and missed the *Rev *completely when it was used to predict genes for the HIV-I virus [14]. Additionally, almost half of the GeneMarkS predictions for influenza genomes picked start codons upstream of the correct start codons.

### Rotavirus

Rotavirus genomes are made up of 11 segments of double stranded RNA encoding 6 viral structural proteins (VP1-4, VP6-7) and 6 non-structural proteins (NSP1-6). Non-structural protein 5 and 6 are encoded by same genomic segment; the coding regions overlap, but are in different reading frames [18]. 19 G types and 27 P types of rotaviruses based on structural proteins VP7 and VP4 were recorded We downloaded from GenBank 1166 rotavirus sequence segments with 1158 annotated genes, and ran in parallel VIGOR, ZCURVE_V and GeneMarkS analyses. VIGOR predicted 1202 protein coding genes including 44 newly detected ORFs which were not annotated in GenBank. Three predictions picked different start codons compared to the annotations in GenBank. These new predictions were examined closely and all of them are homologous to NSP6 with very good similarities (E-value < 1e-10, data not shown).

ZCURVE_V performed well for rotavirus genome gene prediction (Table 1). 1112 of the 1171 predictions were the same as the annotations in GenBank. Both the specificity and sensitivity are approximately 95%; only one protein coding gene was not picked by ZCURVE_V, and 45 were predicted with different start codons. The performance of GeneMarkS was limited for rotavirus gene prediction. Approximately 64% of the predictions (776 predictions) selected the wrong start codons (Table 1).

### Rhinovirus

The rhinovirus genome encodes one polyprotein precursor which is cleaved into eleven functional mature peptides [20]. Thirty-six annotated rhinovirus genomes were downloaded from GenBank and tested with VIGOR, GeneMarkS and ZCURVE_V. VIGOR correctly predicted the polypeptide start codon and stop codon, as well as the mature peptides for each genome (see Table 2). GeneMarkS identified all 36 polyproteins, but predicted the wrong start codons for four genomes. An additional nine small ORFs were incorrectly predicted in the 5'-UTR. ZCURVE_V predicted 77 genes in total, including the 36 true ORFs and 41 mis-predicted small peptides. The start codons of 6 real open reading frames were not correctly predicted.

**Table 2:** Comparative analysis of the annotations in GenBank and predictions by VIGOR, GeneMarkS and ZCURVE_V of RNA viruses, coronaviruses (CoV), SARS coronaviruses (SARS) and rhinoviruses (Rhv)

		**No. of gen**.	No. of genes	**No. of correct pred**.	Sp(%)/Sn(%)	No. of partial correct genes	No. of missing genes	No. of mis-Pred. genes	No. of new genes	**Pred. mat. pep**.
	Ref. Seq.	38	341							
	
	VIGOR		354	353	99.72/99.44	1	1	0	14	No
CoV										
	GeneMarkS		314	247	78.66/69.58	53	48	14	7	No
	
	ZCURVE_V		339	256	75.52/72.11	50	34	26	7	No

	Ref. Seq.	102	1322							
	
	VIGOR		1447	1447	100/99.93	0	1	0	127	Yes
SARS										
	GeneMarkS		941	701	74.50/48.41	119	523	121	21	No
	
	ZCURVE_V		1204	1034	85.88/71.41	107	257	63	76	No

	Ref. Seq.	36	36							
	
	VIGOR		36	36	100/100	0	0	0	0	Yes
Rhv										
	GeneMarkS		45	32	71.11/88.89	4	0	9	0	No
	
	ZCURVE_V		77	30	38.96/83.33	6	0	41	0	No

VIGOR was also used to predict the polyproteins and mature peptides for 66 ATCC rhinovirus samples and field samples sequenced at JCVI with 100% specificity and sensitivity [21]. Neither GeneMarkS nor ZCURVE_V was designed to predict mature peptide sequences for viral genomes.

### Coronavirus

Coronavirus genomes are 27 to 31 Kb in size and encode 9-15 proteins. The genomic structure of each species in the coronavirus genus is highly variable [6] with considerable species diversity among the non-structural proteins. The first open reading frame occupies about 2/3 of the genome, and ribosomal slippage occurs in the expression of this transcript, producing two polypeptides (orf1a and orf1ab) which are cleaved into functional mature peptides. Coronavirus genomes also encode overlapping genes and genes which are completely embedded within other genes.

To evaluate the performance of VIGOR for coronavirus gene prediction, 38 annotated complete coronavirus genomes containing annotation for 341 genes were downloaded from GenBank and run through VIGOR, GeneMarkS and ZCURVE_V. VIGOR identified 354 ORFs, while GeneMarkS and ZCURVE_V predicted 314 and 339 protein coding genes respectively (Table 2). Of the 341 GenBank annotated genes, VIGOR correctly predicted 339 genes, missed one gene and identified one gene with wrong start codon (Table 2). VIGOR also predicted 14 new ORFs which were not annotated in GenBank. Manual curation of these 14 newly predicted proteins showed that they are highly similar (E value < 1e-10) to annotated coronavirus or other viral proteins (data not shown); thus we classified these 14 newly identified genes in coronaviruses as correct predictions. Of the 341 annotated genes, GeneMarkS and ZCURVE_V did not detect 48 and 34 genes respectively. Most of the missing genes were short overlapping genes. The small structural envelope protein coding gene in 10 coronavirus genomes was not identified by either of these two programs because the coding region of this envelope protein overlaps with the coding region of an upstream gene.

VIGOR was also evaluated and used successfully for the gene prediction of more than 50 coronavirus genomes sequenced at JCVI; the specificity and sensitivity were greater than 99% [22-25].

VIGOR has been adjusted as well to optimally predict the protein coding genes in SARS coronavirus genomes. We downloaded from GenBank 102 annotated SARS coronavirus genomes, containing a total of 1322 annotated genes. VIGOR, GeneMarkS, and ZCURVE_V were run for these SARS coronavirus genomes to identify protein coding genes. VIGOR detected 1447 ORFs, 1321 of which completely agreed with the annotations in GenBank (Table 2). Only one GenBank annotated gene was missing on the VIGOR prediction list. VIGOR also found 126 ORFs in these SARS coronavirus genomes which were not annotated in GenBank. By searching the NCBI NR database, the similarity search showed that these 126 newly detected genes encode proteins highly similar (E value < 1e-10) to proteins in SARS coronavirus or other viruses.

ZCURVE_V predicted 1204 genes, 958 of which were identical to the annotations in GenBank. One hundred seven ZCURVE_V predictions have different start codons compared to the annotations in GenBank (Table 2). This program also detected 76 new ORFs which did not exist in GenBank; as with VIGOR, the encoded proteins are highly similar to other viral proteins in GenBank (data not shown). Sixty-three predictions may be incorrect since they could not be corroborated by similarity searches. These were either small peptides (shorter than 50 aa) or were located within the first long open reading frame.

GeneMarkS detected only 941 genes and 680 of them were precise predictions. One hundred nineteen predictions hit the correct regions with the correct frame but the start codons were incorrect (Table 2). GeneMarkS also picked 21 new ORFs which are similar to other viral proteins. One hundred twenty-one GeneMarkS predictions may be incorrect since no homologous proteins could be found in GenBank. Two hundred fifty-seven and 523 GenBank annotations were missing on the ZCURVE_V and GeneMarkS prediction list. These missing genes were examined closely, and most of them are overlapping genes, such as, non-structural protein 3b, 9b, envelope protein, or the gene encoding a non-structural protein which is completely embedded within the coding region of nucleocapsid protein.

The gene predictions from two SARS coronavirus genomes (NC_009695 and AY485277) are detailed in Table 3. NC_009695 is the genomic sequence of a bat SARS-like coronavirus published in 2005 [26]. The annotations have been updated several times by different annotators. This genome encodes 14 ORFs in GenBank. VIGOR predicted 13 ORFs and detected one mutation which resulted in a truncated non-functional peptide (Table 3). The 13 predicted ORFs were exactly the same as the annotations in GenBank. The mutation detected by VIGOR is located in orf3b and generates an internal stop codon, creating truncated peptide of 114 amino acids. The orf3b gene in other coronaviruses is ~154 aa long. We believe this truncated protein is non-functional. ZCURVE_V identified 11 ORFs but missed the two short ORFs (28094-28387, 28544-28756) which are completely embedded in the coding region of the nucleocapsid protein. GeneMarkS detected 8 ORFs but ignored 3 additional ORFs. One was the envelope protein gene (26056-26286), and the other two were non-structural protein genes (27573-27707, 27713-28082). Both GeneMarkS and ZCURVE_V did not predict the orf1ab correctly.

**Table 3:** Comparison of the annotations in GenBank and predictions by VIGOR, GeneMarkS and ZCURVE_V of two SARS coronavirus genomes, NC_009695 (NC) and AY485277 (AY)

	Annotations in GenBank		Predictions by VIGOR		Predictions by GeneMarkS		Predictions by ZCURVE_V	
	
	start	stop	start	stop	start	stop	start	stop
	261	13394	261	13394	261	13394	261	13394
	
	13379	21466	13379	21466	***13580***	21466	***13580***	21466
	
	21473	25198	21473	25198	21473	25198	21473	25198
	
	25207	26031	25207	26031	25207	26031	25207	26031
	
	25628	25972	mutation	mutation				
	
	26056	26286	26056	26286			26056	26286
	
NC	26333	26998	26333	26998	26333	26998	26333	26998
	
	27009	27200	27009	27200	27009	27200	27009	27200
	
	27208	27576	27208	27576	27208	27576	27208	27576
	
	27573	27707	27573	27707			27573	27707
	
	27714	28082	27714	28082			27714	28082
	
	28084	29349	28084	29349	28084	29349	28084	29349
	
	28094	28387	28094	28387				
	
	28544	28756	28544	28756				

	265	13413	265	13413	265	13413	265	13413
	
	265	21485	265	21485	***13599***	21485	***13599***	21485
	
	21492	25259	21492	25259	21492	25259	21492	25259
	
	25268	26092	25268	26092	25268	26092	25268	26092
	
	25689	26153	25689	26153				
	
	26117	26344	26117	26344			26117	26344
	
AY	26395	27060	26395	27060	26395	27060	26395	27060
	
			**27071**	**27262**	**27071**	**27262**	**27071**	**27262**
	
			**27270**	**27638**	**27270**	**27638**	**27270**	**27638**
	
			**27635**	**27769**			**27635**	**27769**
	
			**27776**	**27895**			**27776**	**27895**
	
			**27861**	**28115**			**27861**	**28115**
	
	28117	29385	28117	29385			28117	29385
	
			**28127**	**28423**	**28127**	**28423**		
	
			**28580**	**28792**	***28420***	***29385***		

Bold coordinates indicate the new, correct predictions. Bold, italic coordinates are incorrect predictions.

AY485277 is another SARS coronavirus genome that has 8 ORFs annotated in GenBank [27]. VIGOR predicted an additional 7 ORFs (Table 3). These 7 ORFs were corroborated by comparing them with other viral proteins in GenBank. ZCURVE_V detected 12 ORFs including 5 ORFs which were not annotated in GenBank; these 5 ORFs are highly homologous to other viral proteins. One ORF annotated in GenBank and two ORFs predicted by VIGOR were ignored by ZCURVE_V. GeneMarkS identified 9 ORFs. Three GenBank annotated genes were missing, and 3 VIGOR predictions were also missing from the GeneMarkS prediction list. Neither GeneMarkS nor ZCURVE_V predicted orf1ab protein correctly.

Two *ab initio *gene prediction programs, ZCURVE_CoV [28] and GeneDecipher [29], were specifically trained and the program parameters were adjusted for SARS coronavirus genomes. Both programs can correctly predict the major large protein coding genes and structural protein coding genes like polyprotein orf1a, orf1ab, spike gene, nucleocapsid gene, envelope gene and membrane protein gene. However, short peptide genes and embedded genes were often missing on the predicted gene list (false negative) [29], although the exact function of these small peptide genes is unknown. Mature peptide prediction is not a designed function for these two programs. 17 of the 18 SARS-CoV genomes tested by GeneDecipher were used to evaluate VIGOR predictions. Since there were no annotations for most of these genomes in GenBank, SARS-CoV genome TOR2 genome was annotated in GenBank and predictions were listed [29], a detail comparison was done for this genome (data not shown). The predictions of VIGOR for SARS-CoV genome TOR2 were exactly same as the GenBank annotations. GeneDeciper didn't pick 6 small non-structural protein genes and predicted one gene incorrectly. The genome structure and genes of the other 16 tested SARS-CoV genomes are same as SARS-CoV genome TOR 2, 14 genes and one non-functional non-structural protein gene were detected in these genomes by VIGOR.

### VIGOR usage in high-throughput viral genome closure and annotation

VIGOR has been used extensively to validate the genomes in the finishing process for the high-throughput virus sequencing projects at JCVI [21-25,30]. In this role, VIGOR is used to detect sequence changes which generate a premature stop codon or a frameshift. The potential sequence error and the flanking sequences as well as the BLASTX alignment results are presented in the prediction output. This data allows researchers working on finishing tasks to investigate whether the sequence changes are due to laboratory error or are biologically relevant SNPs or IN/DELs. We noticed that if a mutation or a sequence error generates a pre-mature stop codon or causes frame-shift, and the translated product still meets all criteria stated above, VIGOR will predict this gene as a functional gene. In this type of cases, VIGOR prediction may not be able to identify the potential sequence errors. However, VIGOR provides the alignment data between the predicted peptide and reference sequence in the output alignment file, users can use the alignment data to evaluate the prediction and identify the potential sequence errors. If a genomic sequence covers only a fraction of a gene coding region, VIGOR will identify this genomic sequence as partial sequence. Genome finishing team is able to pursue finishing the genome basing the missing regions identified by VIGOR.

VIGOR has also been used in the gene annotation and submission process. One of the advantages of VIGOR is that it can be used on large numbers of viral genomes simultaneously. The efficiency of VIGOR varies depending on the viral sequence type used as input. For example, using four hundred and fifty eight influenza genomes (6 Mb in total of nucleotide sequences) VIGOR took 85 minutes to complete the gene predictions. In comparison, it took VIGOR 23 minutes to execute the gene prediction for 102 SARS coronavirus genomes (3 Mb in total nucleotide sequences).

## Conclusion

We have demonstrated that VIGOR, a RNA virus gene prediction tool, can predict protein coding genes with high accuracy for 5 different RNA virus types, influenza virus, rotavirus, rhinovirus, coronavirus and SARS coronavirus. VIGOR is available for public use at http://www.jcvi.org/vigor. VIGOR has been thoroughly field tested in several high throughput genome sequencing projects at the JCVI. VIGOR has been employed to predict the protein coding genes successfully for 51 newly sequenced group A rotavirus complete genomes sequenced at JCVI [30] and to annotate and predict mature peptides for 66 rhinovirus full genome sequences [21]. The similarity based program has been also used to annotate the published sequences of bovine, feline, human, murine, rat, SARS and several novel wild animal coronavirus genomes [22-25]. Partial genomes and the potential sequence errors which generate pre-mature stop codons or frameshifts were identified by VIGOR as well during the genome finishing process for these viral sequencing projects.

VIGOR detects protein coding sequences based on similarity searches in conjunction with the known genome specific features for the particular viral genomes. Genes with introns, overlapping genes, and even the genes with a frameshift due to ribosomal slippage can be identified accurately because VIGOR includes these complex mechanisms in the processing for the designated genomes. Both the specificity and sensitivity of VIGOR for the tested genomes was greater than 99%. The same sets of viral genomes were tested for two existing universal viral gene predication methods, the specificity was between 31% and 95%, and the sensitivity was from 35% to 96%. VIGOR was designed to predict the mature peptides accurately for rhinovirus genomes and SARS coronavirus genomes, which is not applicable for the existing universal gene prediction tools. VIGOR can also conduct genotyping and assign function to the predicted protein, both of which are not capable for most available viral gene prediction tools. This user-friendly program is convenient for high throughput sequencing projects and for use by individual laboratories. If reference protein sequences can be collected, and genome specific features are added to VIGOR, this program can extend its capability to predict the protein coding genes in many other small viral genomes.

## Availability and requirements

◦ Project name: VIGOR

◦ Project home page: http://www.jcvi.org/vigor

◦ Operating System(s): Platform-independent

◦ Programming Language: Perl and PHP

◦ Any restrictions to use by non-academics: None.

## Authors' contributions

SW designed and implemented the gene prediction programs, also helped with implementation of the web service back-end infrastructure, evaluated VIGOR, and drafted this manuscript. DS Designed this project and refined this manuscript. JPS designed and implemented the interface and web service back-end infrastructure and refined the manuscript. All authors read and approved the final manuscript.
